# MiR-140 leads to MRE11 downregulation and ameliorates oxaliplatin treatment and therapy response in colorectal cancer patients

**DOI:** 10.3389/fonc.2022.959407

**Published:** 2022-10-17

**Authors:** Josef Horak, Alexandra Dolnikova, Ozge Cumaogullari, Andrea Cumova, Nazila Navvabi, Ludmila Vodickova, Miroslav Levy, Michaela Schneiderova, Vaclav Liska, Ladislav Andera, Pavel Vodicka, Alena Opattova

**Affiliations:** ^1^ Department of Molecular Biology of Cancer, Institute of Experimental Medicine Czech Academy of Sciences (CAS), Prague, Czechia; ^2^ Third Faculty of Medicine, Charles University, Prague, Czechia; ^3^ First Faculty of Medicine, Charles University, Prague, Czechia; ^4^ Eastern Mediterranean University, Dr. Fazıl Küçük Faculty of Medicine, North Cyprus, Turkey; ^5^ Gazimağusa State Hospital, Molecular Genetics Research Laboratory, North Cyprus, Turkey; ^6^ Biomedical Center in Pilsen, Charles University, Pilsen, Czechia; ^7^ Surgical Department, 1.st Medical Faculty, Charles University and Thomayer Hospital, Prague, Czechia; ^8^ Department of Surgery, University Hospital Kralovske Vinohrady and 3rd Faculty of Medicine, Charles University, Prague, Czechia; ^9^ Department of Surgery, Medical Faculty in Pilsen, Charles University, Pilsen, Czechia; ^10^ Institute of Biotechnology, Czech Academy of Sciences (CAS), Vestec, Czechia

**Keywords:** miR-140, colorecal cancer, *MRE11*, oxaliplatin, therapy response, DNA damage, DNA repair, miRNA

## Abstract

Cancer therapy failure is a fundamental challenge in cancer treatment. One of the most common reasons for therapy failure is the development of acquired resistance of cancer cells. DNA-damaging agents are frequently used in first-line chemotherapy regimens and DNA damage response, and DNA repair pathways are significantly involved in the mechanisms of chemoresistance. MRE11, a part of the MRN complex involved in double-strand break (DSB) repair, is connected to colorectal cancer (CRC) patients’ prognosis. Our previous results showed that single-nucleotide polymorphisms (SNPs) in the 3′ untranslated region (3′UTR) microRNA (miRNA) binding sites of *MRE11* gene are associated with decreased cancer risk but with shorter survival of CRC patients, which implies the role of miRNA regulation in CRC. The therapy of colorectal cancer utilizes oxaliplatin (oxalato(trans-l-1,2-diaminocyclohexane)platinum), which is often compromised by chemoresistance development. There is, therefore, a crucial clinical need to understand the cellular processes associated with drug resistance and improve treatment responses by applying efficient combination therapies. The main aim of this study was to investigate the effect of miRNAs on the oxaliplatin therapy response of CRC patients. By the *in silico* analysis, miR-140 was predicted to target MRE11 and modulate CRC prognosis. The lower expression of miR-140 was associated with the metastatic phenotype (p < 0.05) and poor progression-free survival (odds ratio (OR) = 0.4, p < 0.05). In the *in vitro* analysis, we used miRNA mimics to increase the level of miR-140 in the CRC cell line. This resulted in decreased proliferation of CRC cells (p < 0.05). Increased levels of miR-140 also led to increased sensitivity of cancer cells to oxaliplatin (p < 0.05) and to the accumulation of DNA damage. Our results, both *in vitro* and *in vivo*, suggest that miR-140 may act as a tumor suppressor and plays an important role in DSB DNA repair and, consequently, CRC therapy response.

## Introduction

Treatment failure of colorectal cancer (CRC) therapy, represented by the development of drug resistance or outgrowth of metastasis, is a major complication for CRC patients. There is a crucial clinical need for predictive biomarkers that indicate the success or failure of cancer treatment. A better understanding of the cellular processes associated with drug resistance will eventually lead to improved treatment response by applying more effective combination therapies (1).

Cancer cells react toward chemotherapeutics in different modes, such as by modifying DNA repair pathways. DNA repair plays a major role in the cancer therapy response, as chemotherapeutics usually induce various types of DNA damage in cancer cells (2). The overexpression of DNA repair genes in the tumor may confer more efficient repair of induced damage and thus contribute to chemoresistance and impaired therapy response (3). However, downregulation of the DNA repair genes may confer a better therapy response but may also give a basis for the appearance of new mutations and cancer progression (4).

Oxaliplatin (oxalato(trans-l-1,2-diaminocyclohexane)platinum; OX) belongs to the most used chemotherapeutics in CRC treatment. OX is a genotoxic drug that induces the formation of DNA crosslinks, thus directly impairing the structure of DNA, inhibiting DNA replication and RNA synthesis, and inducing apoptosis (5). One of the most crucial repair pathways to deal with DNA crosslinks is homologous recombination (HR), a constituent of double-strand break (DSB) repair (6).

MRN complex, a protein complex consisting of MRE11-RAD50-NBS1, plays an important role in the initial processing of DSB repair. The impaired function of the MRN complex leads to gene instability and DNA damage accumulation, a prerequisite of malignant transformation (7). Mutations in *MRE11* predispose to CRC and are frequent in primary CRC with mismatch repair deficiency (8). Patients with the decreased expression of MRE11 were more sensitive to OX treatment, with more significant tumor mass reduction and more prolonged progression-free survival (9). Moreover, single-nucleotide polymorphisms (SNPs) in the 3′ untranslated region (3′UTR) of *MRE11* gene are associated with decreased cancer risk but with shorter survival in CRC patients, which implies the role of microRNA (miRNA) regulation in CRC (10).

MiRNAs are signaling molecules in various cell processes functioning mainly as the suppressors of gene expression through interaction with 3′UTRs of target mRNAs. However, miRNAs have also been shown to interact with other regions of mRNA and can even activate gene expression under certain conditions (11). There are several mechanisms by which the deregulation of miRNAs can influence malignant transformation (for review, see (12)). Regardless of the mechanism, miRNA dysregulation can potentiate CRC development by acquiring one or more hallmarks of cancer (13). Despite some evidence of miRNAs influencing the CRC sensitivity to the therapy, there is a scarcity of miRNAs associated with OX therapy response (14).

The main aim of this study was to investigate the effect of miRNAs on the OX therapy response of CRC patients. Based on our previous published study, where we observed an association of SNPs in the 3′UTR of the *MRE11* gene with decreased CRC risk (10), we performed *in silico* analysis of miRNAs associated with MRE11 and found 187 miRNAs with *MRE11* as a predicted target. By additional analysis using The Cancer Genome Atlas (TCGA) database, we have identified miR-140 as the best candidate for further investigation. Our results suggest that the miR-140/MRE11 axis is associated with improved therapeutic response in oxaliplatin-treated CRC patients.

## Materials and methods

### Patient characteristics and samples

Paired tumor and non-malignant adjacent mucosa samples were obtained from 50 patients who underwent surgery between the years 2011 and 2015 and in whom all information was followed and updated in 2021 (patients’ characteristics in **Table 1** and **Supplementary Table 1**). All the patients provided signed consent for participation and their medical documentation for research. The design of the study was approved by the Ethical Committee of the Institute of Experimental Medicine, Prague, Czech Republic. RNA was isolated from tissues by miRNeasy^®^ Mini Kit (50) (Qiagen, Hilden, Germany).

**Table 1:** Patients’ characteristics.

		Number of patients (N = 50)
Gender, N	Male	26
	Female	24
Age of diagnosis	Median	65
	Range	37-82
Smoker, N	Smokers	16
	Non-smokers	16
	Ex-smokers	18
TNM stage, N	I	2
	II	13
	III	25
	IV	10
Metastasis	Yes	26
	No	24

### Bioinformatics analysis

Data from TargetScan (15) were extracted by multiMiR R package (16).

All miRNA-Seq transcriptional profiles and detailed clinical information were downloaded from TCGA (https://portal.gdc.cancer.gov) using the TCGAbiolinks R package (17). For the present study, data from the project TCGA-READ (rectal adenocarcinoma, n = 155) and TCGA-COAD (colon adenocarcinoma, n = 476) for every miRNA were separately analyzed and filtered according to the following criteria: 1) analyses were performed on CRC patients who had miRNA expression level data available, and 2) clinical data including survival data were also available. Finally, for miR-140, a total of 570 patients presented expression levels.

### Cell cultures

Human colorectal cancer cell lines HCT116, DLD1, and HT29 were obtained from Merck (Darmstadt, Germany). Cell lines were cultured in Dulbecco’s modified Eagle’s medium (DMEM) (Merck, Germany) with 10% fetal bovine serum (Merck, Germany), 1 mM of l-glutamine (Biosera, Nuaille, France), 1 mM of sodium pyruvate (Biosera, Nuaille, France), and 1 mM of penicillin/streptomycin (Biosera, Nuaille, France). All cells were cultured in a humidified incubator at 37°C, with 5% CO2.

### Transient transfection

Cells were transfected in 6-well plates at 60%–80% confluency with 2.5 pmol of MISSION miRNA hsa-miR-140-3p miRNA Mimics (Ambion, Austin, TX, USA) or with Negative Control miRNA Mimics (Ambion, USA) with no homology to the human genome using Lipofectamine^®^ RNAiMAX 2000 (Invitrogen™) according to the manufacturer’s protocol. All the experiments in cell lines were performed in three independent repeats. The efficiency of transfection was analyzed by qPCR measuring expression levels of transfected miRNAs as compared to negative controls.

### Isolation and reverse transcription of RNA from cell culture samples

Forty-eight hours after transfection, total RNA (including miRNAs) was extracted from cells using Qiagen miReasy Mini Kit (Qiagen, Germany) according to the manufacturer’s protocol. The concentration of the total RNA was measured by Nanodrop™ 8000 Spectrophotometer (Thermo Fisher Scientific, Waltham, MA, USA), and the integrity of mRNA (RNA integrity number (RIN)) of each sample was determined by Agilent RNA 6000 Nano Kit by Agilent Bioanalyzer 2100 (Agilent Technologies, Santa Clara, CA, USA). Reverse transcription was performed using the High-Capacity cDNA Reverse Transcription Kit (Thermo Fisher Scientific, USA), according to the manufacturer’s protocol.

### Quantitative PCR of cell culture samples

Expression levels of miR-140 were measured using TaqMan MicroRNA Assays at 7500 Real Time PCR System (Thermo Fisher Scientific, USA). The reaction contained 2 μl of a sample with 40 ng of cDNA, 10 μl of TaqMan™ Universal PCR Master Mix, 1 μl of the assay, and 7 μl of RNAse-free water. The thermal protocol was as follows: 50°C for 2 min, 95°C for 10 min, 40 cycles of 95°C for 15 s, and 60°C for 60 s plus melting curve analysis. MiRNA expression was normalized to RNU6B, and all data were subsequently analyzed by the 2−ΔΔCt method.

### Oxaliplatin treatment

Oxaliplatin, obtained from Merck (Germany), was dissolved in dimethyl sulfoxide (DMSO; Merck, Germany) at the concentration of 100 mM and stored at 4°C. To assess the chemosensitivity of CRC cells with overexpressed miR-140 and control cells, both cells were treated with a 6 μM concentration of oxaliplatin 24 h after miRNA mimics transfection and analyzed for cell viability.

### Viability and proliferation assays

For clonogenicity formation assay (CFA), 48 h after cell transfection with miRNA mimics, 500 cells per well were plated for colony formation assay onto 6-well plates and cultured in DMEM. Twelve days later, colonies were fixed with 3% formaldehyde, stained with 1% crystal violet, and counted.

For proliferation assay, cells were plated onto 96-well plates at a density of 3 × 104 cells per well. The metabolic activity of the cells was measured 24 h after plating by adding WST-1 solution into the media as recommended by the manufacturer (Merck, Germany). Absorbance at 450 and 690 nm was measured on BioTek ELx808 absorbance microplate reader (BioTek, Winooski, VT, USA).

### Cell cycle analysis

Cells were seeded on 12 well plates (5 × 105 cells/ml), harvested, washed with PBS, and centrifuged at 1,000 rpm for 10 min. Then, 1 ml of propidium iodide (PI) staining solution (0.02 µg/µl of PI, 0.02 mg/ml of RNase, and 0.05% Triton X-100) was added to the cell pellet, and cells were incubated for 30 min at 37°C in the dark. After incubation, samples were analyzed using a flow cytometer (Apogee A-50 micro, Apogee, Hertfordshire, UK). Measured data were evaluated with FlowLogic software (Inivai Technologies, Mentone, VIC, Australia).

### Sodium dodecyl sulfate–polyacrylamide gel electrophoresis and Western blotting analysis

Proteins (20 μg) were loaded and separated in 10% sodium dodecyl sulfate–polyacrylamide gel electrophoresis (SDS-PAGE) gels at 15 mA for 60 min. Then, the separated proteins were transferred to 0.45 µm Amersham Protran Nitrocellulose Blotting Membrane (GE Healthcare, Life Sciences, Marlborough, MA, USA) in methanol transfer buffer using Mini Trans-Blot Cell (Bio-Rad Laboratories, Hercules, CA, USA). The membranes were blocked with 5% bovine serum albumin (BSA) in Tris-buffered saline containing Tween 20 (TBST; 20 mM of Tris–HCl, pH 7.4, 0.15 M of NaCl, and 0.1% Tween 20) for 1 h and incubated with anti-MRE11, anti-γH2AX, anti-RAD51 (Cell Signaling, Leiden, the Netherlands) and anti-GAPDH antibodies (Abcam, Cambridge, UK) at 4°C overnight, followed by incubation with goat anti-rabbit secondary antibody conjugated with horseradish peroxidase (Abcam, Cambridge, UK). The membranes were then incubated with Immobilon Western Chemiluminescent HRP Substrate (EMD Millipore Corporation, Billerica, MA, USA) and visualized by Azure c600 (Azure Biosystems, Dublin, CA, USA).

### Preparation and application of recombinant lentiviruses for MRE11 silencing

For the preparation of recombinant lentiviruses expressing MRE11 shRNAs, HEK293FT cells (Thermo Fisher, Waltham, MA, USA) seeded in 6-well plates were co-transfected with pLKO1 mission MRE11 shRNA plasmids and helper plasmids psPax2 and pMD2.g (Addgene, Cambridge, MA, USA) using Lipofectamine 3000 (Thermo Fisher, Massachusetts, USA). Six hours later, the medium was replaced with fresh DMEM without antibiotics. After 48 h, the recombinant lentivirus-containing culture medium was harvested and centrifuged at 15 min, 3,000 rpm, and 4°C to remove any floating cells and cell debris. The cleared media containing lentiviruses were at 1:3 and 1:10 v/v ratios, added to HCT116 cells and plated in a 12-well plate, and after 24 h; the media were replaced with the fresh cultivation medium; cell cultures containing integrated lentiviruses were selected by using 2 μg/ml of puromycin for 4–5 days. Transfected cells were then tested using genomic PCR and Western blotting analysis for the genetic elimination/loss of expression of the *MRE11* gene.

### Statistical analysis

Statistical analyses were performed using pairwise comparison by Student’s t-test and two-way ANOVA (GraphPad Prism8, GraphPad Software, La Jolla, CA, USA; www.graphpad.com). The results represent the mean value of three independent experiments ± SD; the significance level was set at p ≤ 0.05. Statistical analysis for TCGA data was performed using the R environment using the dplyr and survival, survminer, and ggplot2 packages. The survival significance was measured by a log-rank test.

## Results

### *In silico* analysis of miRNAs targeting MRE11

**Figure 1:**
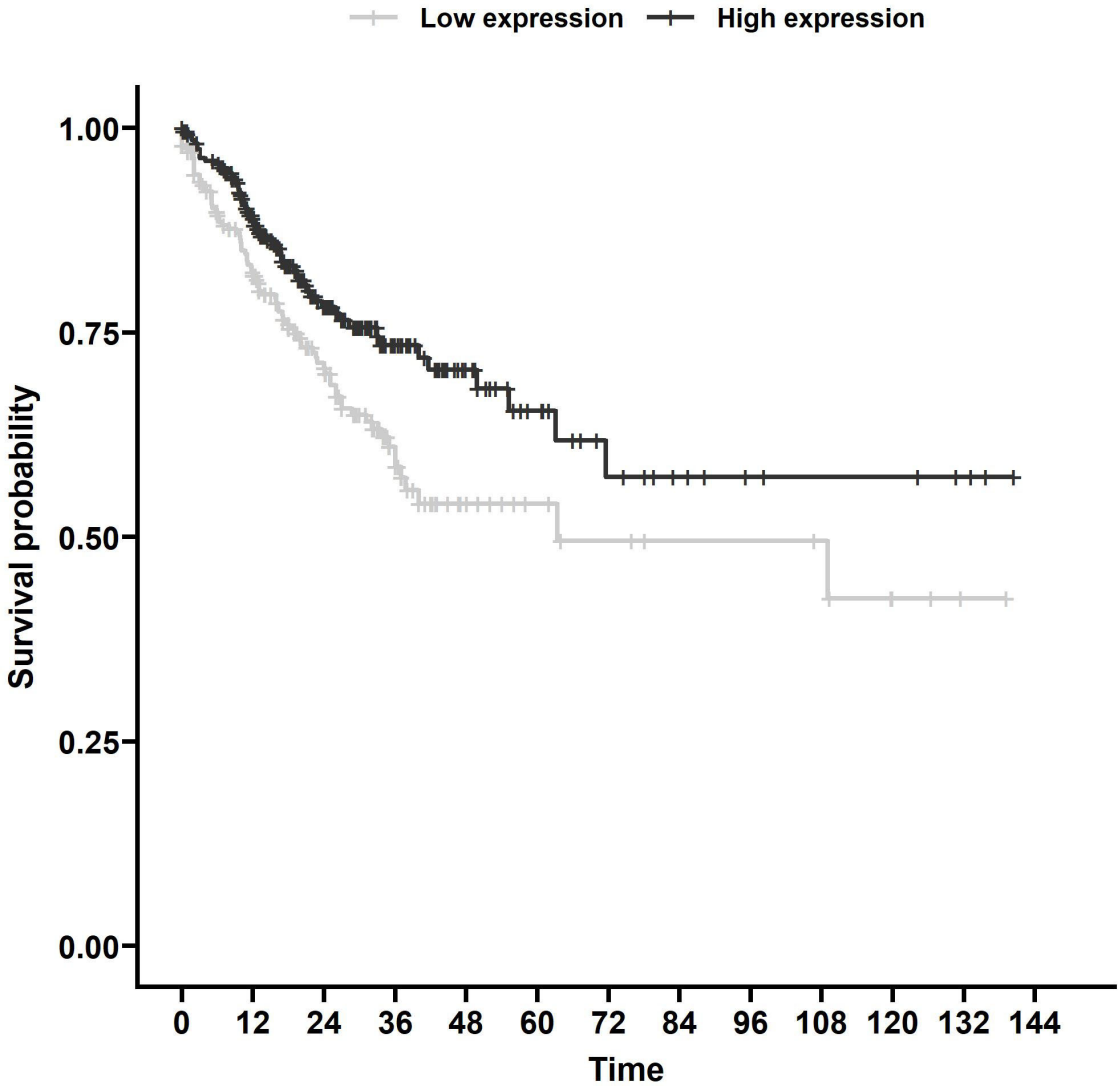
Survival analysis of TCGA samples. PFS associated with miR-140 expression of CRC patients from TCGA database. Kaplan–Meier analysis of the TCGA dataset showed that lower miR-140 expression is associated with poor PFS (p = 0.006). TCGA, The Cancer Genome Atlas; PFS, progression-free survival; CRC, colorectal cancer.

Using TargetScan (15), we found 187 miRNAs with *MRE11* as a predicted target (**Supplementary Table 2**) with 111 miRNAs with data sufficient for progression-free survival (PFS) calculation in the TCGA database. Out of these 111 miRNAs, eight had a statistically significant impact on PFS (p < 0.05, **Supplementary Table 3**). We identified miR-140 as the candidate for further investigation, as it displayed the strongest statistically significant association with PFS (**Figure 1**, p < 0.01) in the group of analyzed miRNAs supported by data from more than 500 patients.

### MiR-140 is downregulated in colorectal cancer and associated with progression-free survival and with the metastatic phenotype in colorectal cancer patients’ samples

**Figure 2:**
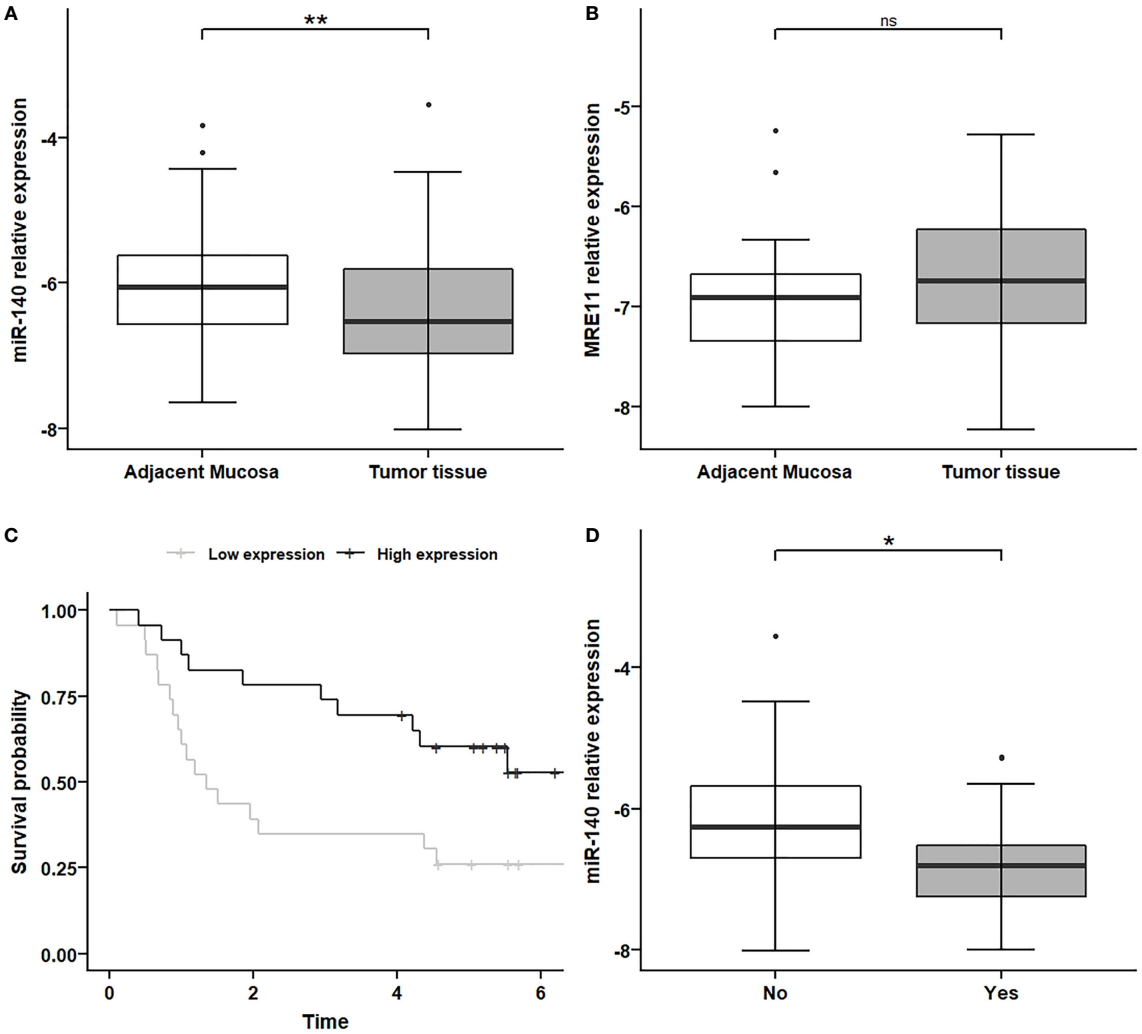
MiR-140 downregulation and association with PFS and metastatic CRC phenotype. **(A)** Relative expression of miR-140 is decreased in tumor tissues compared to non-adjacent mucosa (p = 0.009). **(B)** Relative expression of MRE11 in non-significantly increased in tumor tissue (p = 0.11). **(C)** Kaplan–Meier analysis showed that lower expression of miR-140 in tumor tissue is associated with poor PFS (p = 0.017). **(D)** Decreased relative expression of miR-140 is associated with the metastatic phenotype of CRC (p = 0.023). *p ≤ 0.05, **p ≤ 0.01. PFS, progression-free survival; CRC, colorectal cancer. ns = p>0.05 = non-significant.

We investigated the expression levels of MRE11 and miR-140 in 50 CRC tumor tissues and adjacent non-malignant mucosa samples (**Table 1** and **Supplementary Table 1**). The levels of miR-140 were significantly lower in tumor tissue (**Figure 2A**, p < 0.01) compared to adjacent mucosa. MRE11 levels were moderately, but not significantly, higher in tumor tissues (**Figure 2B**, p = 0.11). A significant decrease in miR-140 in patients’ CRC samples led only to a moderate non-significant increase in MRE11, which might be due to broader regulation, mixed phenotype, or complex treatment.

The Kaplan–Meier analysis showed, in concordance with TCGA results, that lower expression of miR-140 in tumor tissue is associated with poor PFS (**Figure 2C**, p < 0.05).

Because metastatic CRC has a higher mortality rate and treatment is much more challenging, we have also investigated the association between miR-140 and metastatic formation. Our data showed that decreased expression of miR-140 is associated with the metastatic phenotype of CRC (**Figure 2D**, p < 0.05).

### MiR-140 represses MRE11 expression

**Figure 3:**
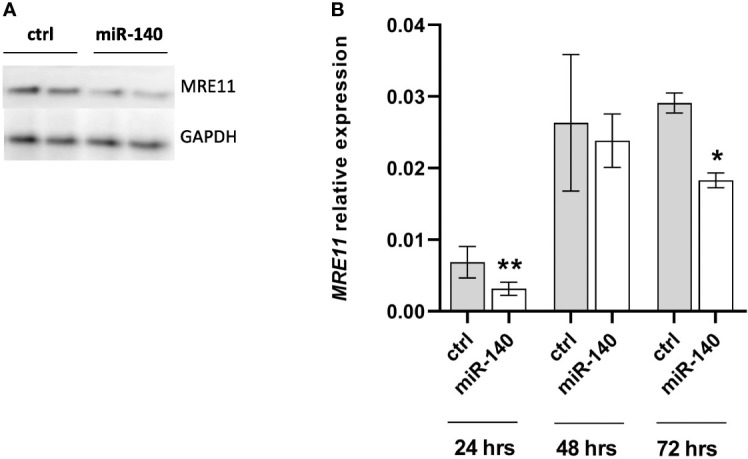
Increased levels of miR-140 led to the downregulation of MRE11. **(A)** Western blotting analysis of cells after transient transfection of miR-140 in the DLD1 cell line showed decreased protein level of MRE11. **(B)** qPCR analysis of cells showed decreased MRE11 mRNA level. The results represent the mean value of three independent experiments ± SD. *p ≤ 0.05, **p ≤ 0.01.

To select the appropriate colorectal cell line for transient transfection, we measured the expression levels of miR-140 in different CRC cell lines (**Supplementary Figure 1A**), and we decided on DLD1 by transient transfection of miR-140 by miRNA mimics. We have reached a significant increase in miR-140 levels stable up to 72 h (**Supplementary Figure 1B**). Our data showed that overexpression of miR-140 using miRNA mimics decreased the protein levels of MRE11 (**Figure 3A**) as well as mRNA levels of MRE11 (**Figure 3B**).

### Overexpression of miR-140 leads to the accumulation of DNA damage

**Figure 4:**
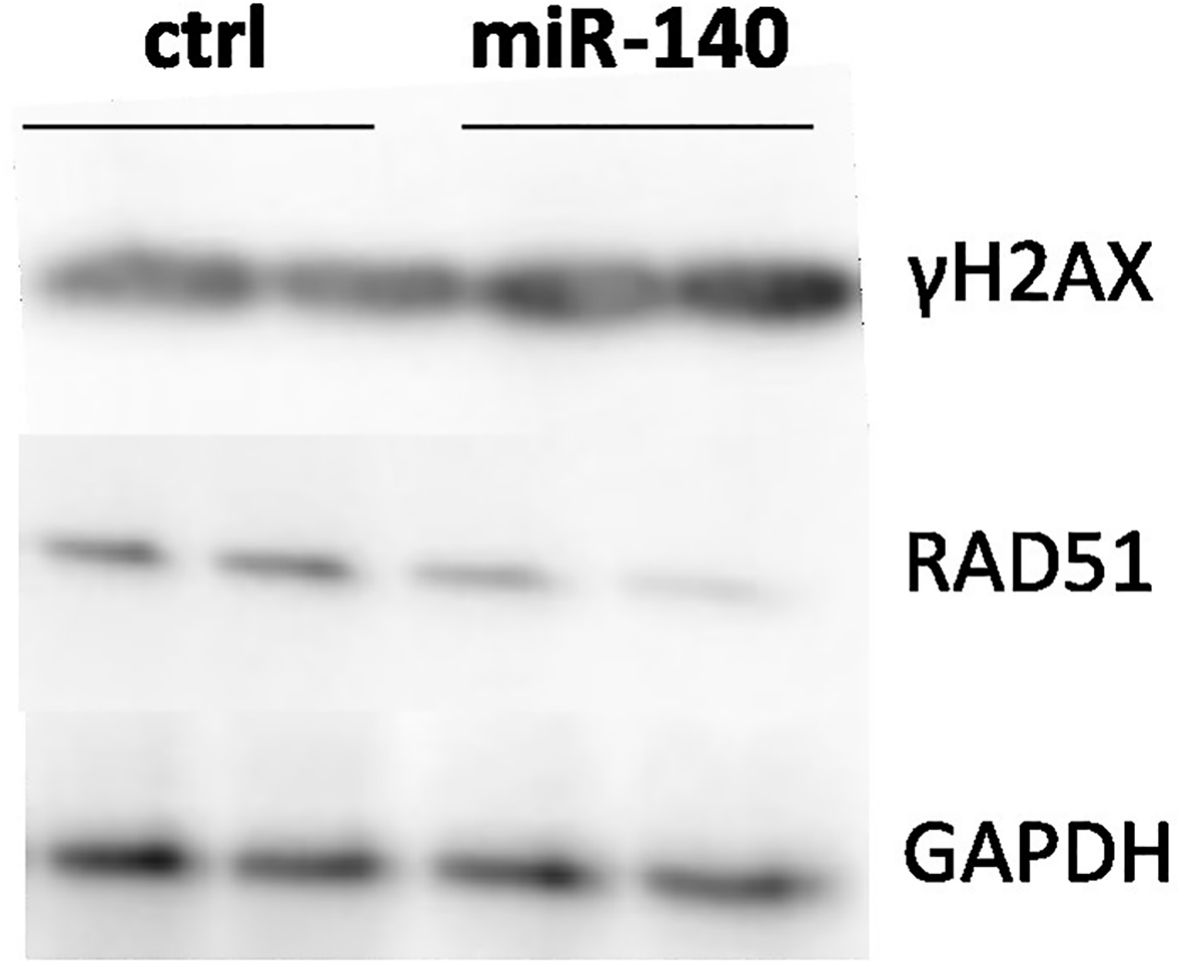
Effect of miR-140 on markers of DSBs. Western blotting analysis of cells transiently transfected with miR-140 showed decreased protein levels of RAD51 and increased levels of γH2AX. DSBs, double-strand breaks.

MRE11 is a crucial component of the MRN complex associated with DSB repair (18). Therefore, we evaluated the effect of miRNA mimic-induced miR-140 overexpression on one of the markers of DSB DNA damage and γH2AX protein accumulation (19). Western blotting analysis showed higher levels of γH2AX after miR-140 miRNA mimics in the CRC cell line (**Figure 4**).

### Overexpression of miR-140 decreases colorectal cancer cell proliferation

**Figure 5:**
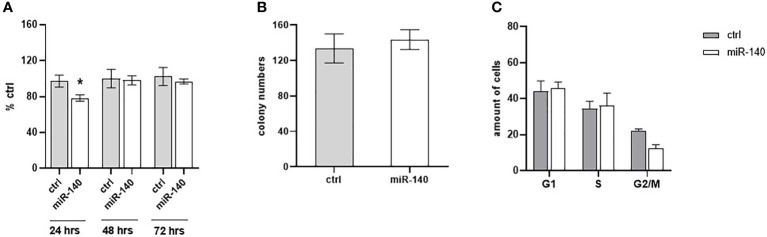
Effect of miR-140 on colorectal cancer cells. **(A)** Proliferation analysis showed a decreased level of proliferation in cells overexpressed miR-140 after 24 h (p = 0.05). **(B)** miR-140 overexpression did not affect the clonogenic potential of the cells. **(C)** Analysis of cell cycle content showed moderate accumulation of cells in the G1 phase. The results represent the mean value of three independent experiments ± SD. *p ≤ 0.05.

The effect of miR-140 overexpression induced by miRNA mimics on CRC cell proliferation was measured using the WST-1 assay. **Figure 5A** shows that overexpression of miR-140 leads to decreased cell proliferation, pronounced 24 h after transfection (p = 0.05). However, miR-140 overexpression does not affect clonogenic potential (**Figure 5B**). In addition, flow cytometry analysis of the cell cycle showed that overexpression of miR-140 leads to moderate accumulation of cells in the G1 phase (**Figure 5C**).

### MiR-140 enhances the chemotherapeutic sensitivity of colorectal cancer cells

**Figure 6:**
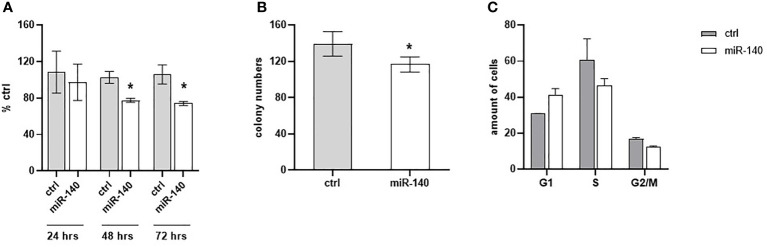
MiR-140 enhances the oxaliplatin sensitivity of CRC cells. **(A)** CRC cell proliferation after miR-140 and oxaliplatin treatment is decreased after 48 h (p < 0.05) and 72 h (p < 0.05). **(B)** Analysis of cell clonogenicity potential showed a significant decrease in CFA (p < 0.05). **(C)** Analysis of cell cycles showed an accumulation of cells in the G1 phase and a decrease in the S phase. The results represent the mean value of three independent experiments ± SD. *p ≤ 0.05. CRC, colorectal cancer; CFA, clonogenicity formation assay.

Oxaliplatin is a third-generation platinum compound with an important role in CRC treatment. Therefore, we have investigated miR-140 in relation to the oxaliplatin sensitivity of CRC cells. Cell proliferation after oxaliplatin treatment in DLD1 cells overexpressing miR-140 significantly decreased after 48 and 72 h (**Figure 6A**, p < 0.05). The clonogenic potential of the cells (CFA) revealed a significant decrease in colony numbers (**Figure 6B**, p < 0.05). Cell cycle analysis of oxaliplatin-treated cells showed that overexpression of miR-140 leads to an increase in cells in the G1 phase and a decrease in those in the S phase (**Figure 6C**).

### MiR-140 did not affect oxaliplatin sensitivity in shMRE11 cell lines

**Figure 7:**
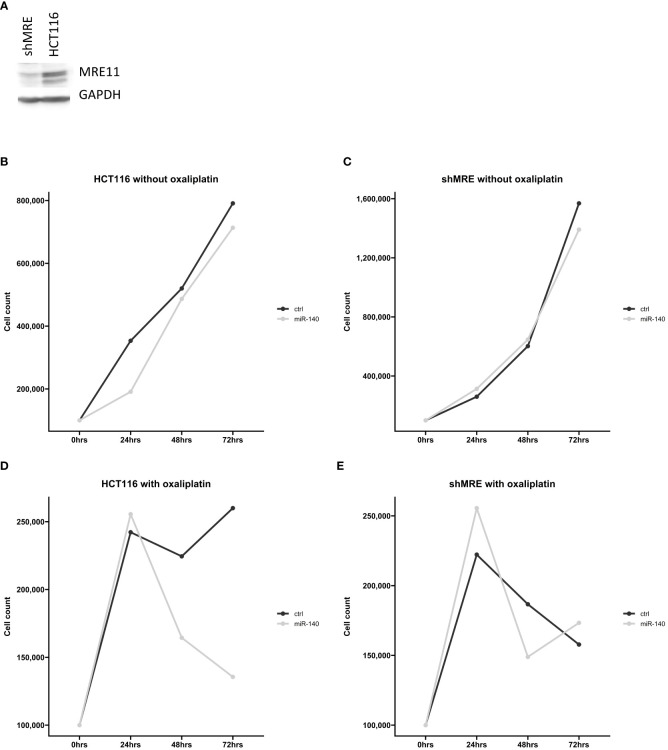
MiR-140 did not affect oxaliplatin sensitivity in shMRE11 cell lines. **(A)** Western blotting analysis of novel established cell line expressing recombinant lentiviruses MRE11 shRNA. **(B)** Cellular growth of parental cells HCT116 after overexpression of miR-140 was not changed. **(C)** Cellular growth of shMRE11 cells after overexpression of miR-140 was not changed. **(D)** Analysis of cellular growth of HCT116 with overexpression of miR-140 and oxaliplatin treatment showed decreased growth (p = 0.05). **(E)** Cellular growth of shMRE11 cells with miR-140 overexpression after oxaliplatin treatment was not changed. The results represent the mean value of three independent experiments.

Our *in silico* analysis proposed a potential connection between miR-140 and MRE11. To further analyze the effect of miR-140 on oxaliplatin sensitivity through MRE11, we used recombinant lentiviruses expressing MRE11 shRNAs and established CRC cell lines with suppressed levels of MRE11 (**Figure 7A**). Cellular growth after miR-140 overexpression was not changed in parental and shMRE11 cell lines (**Figures 7B, C**). The measurement of cellular growth of HCT116 with overexpression of miR-140 and oxaliplatin treatment showed decreased cellular growth (p = 0.05) (**Figure 7D**). However, the analysis of cell growth did not show increased oxaliplatin sensitivity of shMRE11 cells with overexpressed miR-140 (**Figure 7E**).

## Discussion

Poor therapy response and chemoresistance pose significant complications in CRC treatment, leading to ineffective therapy, tumor progression, metastasis, relapse of disease, and impaired patient survival.

Based on our previous evidence that miRSNPs in the *MRE11* gene influence CRC risks and survival (10), in the present study, we investigated the effect of the miRNA/MRE11 axis on the oxaliplatin therapy response of CRC patients.

Despite the multidisciplinary approach and chemotherapy improvement, there is a considerable percentage of patients with inadequate response to treatments and a poor prognosis. Currently, there is a lack of properly validated predictive factors for CRC treatment response, and the emergence of resistant clones is a non-negligible reason for therapeutic failure and potential metastasis development (20). In our study, we defined the association of miR-140 expression with PFS, where lower miR-140 expression is associated with poor survival. Furthermore, our results showed lower levels of miR-140 in tumor tissue. MiR-140 expression has been previously studied mainly in association with cancer development and recurrence. Zheng et al. performed a meta-analysis and found a strong correlation between high expression of miR-140 and better overall survival (OS) in several cancers. Conversely, low expression is associated with advanced stages, worse histologic type, and lymph node metastasis (21). MiR-140 could also remarkably reduce the tumor size in gastric cancer xenograft mice (22). Yuan et al. found that miR-140 is significantly downregulated in non-small lung carcinoma (NSCLC) tissues and cell lines (23). In recent years, there has been increasing evidence of a miR-140 role in the response to platinum derivative treatment in different cancers. Meng et al. described that miR-140 promoted autophagy mediated by HMGN5 and sensitized osteosarcoma cells to chemotherapy (24). Furthermore, miR-140 acts as a tumor suppressor in breast cancer by inhibiting FEN1 from repressing DNA damage repair. The authors of the published work reveal miR-140 to be a new anti-tumorigenesis factor for adjuvant breast cancer therapy (25). These results suggest a therapeutic potential of miR-140 in cancer treatment. Lui et al. demonstrated that plasma exosomal miR-140 in CRC patients was lower than in healthy controls, and their work supports our findings that miR-140 exerts a tumor suppressor ability (26).

Moreover, we found that decreased expression of miR-140 was associated with metastatic CRC phenotype. Our findings are consistent with a study by Shahabi et al. (2020). The authors showed that low expression of miR-140 is associated with lymph node metastasis in breast cancer (27).

Our *in vitro* analysis revealed an association of miR-140 overexpression with decreased CRC cell survival and accumulation of DNA damage. Moreover, overexpression of miR-140 enhances the sensitivity of colorectal cells to oxaliplatin. The important role of miRNA in oxaliplatin resistance in CRC was also proven by Wang et al. (28). They published evidence that overexpression of miR-29b re-sensitized OR-SW480 cells to oxaliplatin treatment. MiR-140 also re-sensitizes cisplatin-resistant NSCLC cells to cisplatin treatment through the SIRT1/ROS/JNK pathway (29).

Direct or indirect induction of DNA damage is the main goal of most cancer treatment regimens. Therefore, the process of DNA damage repair plays an important role in therapy response and chemotherapy resistance. Unfortunately, cancer cells can initiate DNA repair, which plays a role in therapy response (3) and chemotherapy resistance (2). The clinical importance of HR for cancer therapy, mainly of MRE11, RAD50, and, NBS, has already been reported (30). According to Pavelitz et al., deficient MRE11 protein is a marker of better prognosis for CRC patients irrespective of treatment in the long term (31). We previously described the significant influence of miRNA binding sites (miRSNPs) in the *MRE11* gene on CRC risks and survival (10). The importance of SNPs in miRSNPs of DNA repair genes has been also described in other types of cancer (32). MiR-140 was predicted as a potential interacting partner for MRE11 by TargetScan (15). *In vitro* overexpression of miR-140 causes the decrease of MRE11 protein levels. We did not observe any effect of miR-140 on cell proliferation and oxaliplatin sensitivity in the cells with inhibited MRE11 (shMRE11). Based on this data, we hypothesize that miR-140 affects oxaliplatin sensitivity in CRC cells *via* MRE11, or miR-140 may cooperate with MRE11 and may affect oxaliplatin sensitivity in tested cells. MRE11 downregulation may lead to impairment of MRN complex and thus to inefficient HR and subsequent damage accumulation (33). That is in accordance with our results, as we observed the accumulation of γH2AX, a marker of DNA damage, following overexpression of miR-140.

Despite intensive research, the efficiency of CRC therapy remains low. Searching for novel prognostic and predictive biomarkers may lead to better therapy responses. The presence of miRNAs in blood plasma gives miRNAs a solid potential to be easily accessible biomarkers. However, their use may be compromised by the interindividual variability of cancer patients and large intratumor heterogeneity. Our results indicate miR-140 as a tumor suppressor and potential predictive biomarker for oxaliplatin treatment. We believe that identifying and validating novel biomarkers will ultimately lead to more personalized cancer therapy and improve the quality of a CRC patient’s life.

## Data availability statement

The raw data supporting the conclusions of this article will be made available by the authors, without undue reservation.

## Ethics statement

This study was reviewed and approved by the Ethical Committee of the Institute of Experimental Medicine, Prague, Czech Republic. The patients/participants provided written informed consent to participate in this study.

## Author contributions

JH, AD, AC, OC, and AO performed the experiments. LA coordinated the cell line establishment. ML, LV, and MS were responsible for the collection of patients’ samples. PV reviewed the manuscript and discussed the results. AO coordinated the study and wrote a manuscript, JH wrote the manuscript. All authors contributed to the article and approved the submitted version.

## Funding

The study was supported by the Grant Agency of Charles University (GAUK 784120), the Czech Science Foundation (20-03997S, 21-27902S, and 21-04607X), the Czech Health Research Council (grants AZV NV18/03/00199), Charles University grant Unce/Med/006, the Charles University Research Fund (Cooperation No. 43—Surgical Disciplines and the Cooperation Program, research area Oncology and Haematology), EFRR [project No. CZ.02.1.01/0.0/0.0/16_019/0000787 “Fighting INfectious Diseases”, awarded by the MEYS CR], and the National Operation Programme: National Institute for Cancer Research LX22NPO05102.

## Acknowledgments

The results shown in the section “*In silico* analysis of miRNAs targeting MRE11” are in part based upon data generated by TCGA Research Network: https://www.cancer.gov/tcga.

## Conflict of interest

The authors declare that the research was conducted in the absence of any commercial or financial relationships that could be construed as a potential conflict of interest.

## Publisher’s note

All claims expressed in this article are solely those of the authors and do not necessarily represent those of their affiliated organizations, or those of the publisher, the editors and the reviewers. Any product that may be evaluated in this article, or claim that may be made by its manufacturer, is not guaranteed or endorsed by the publisher.

## Supplementary material

Supplementary Figure 1
**(A)** relative expression of miR-140 in different colorectal cell lines **(B)** optimization of the transfection with miR-140 mimics. The results represent the mean value of three independent experiments ± SD *p ≤ 0.05, **p ≤ 0.01, ***p ≤ 0.001
