# Supplementary figures and images for: MiR-140 leads to MRE11 downregulation and ameliorates oxaliplatin treatment and therapy response in colorectal cancer patients
Source: Front Oncol. 2022 Oct 17;12:959407. doi: 10.3389/fonc.2022.959407 (PMC9618941; doi:10.3389/fonc.2022.959407)

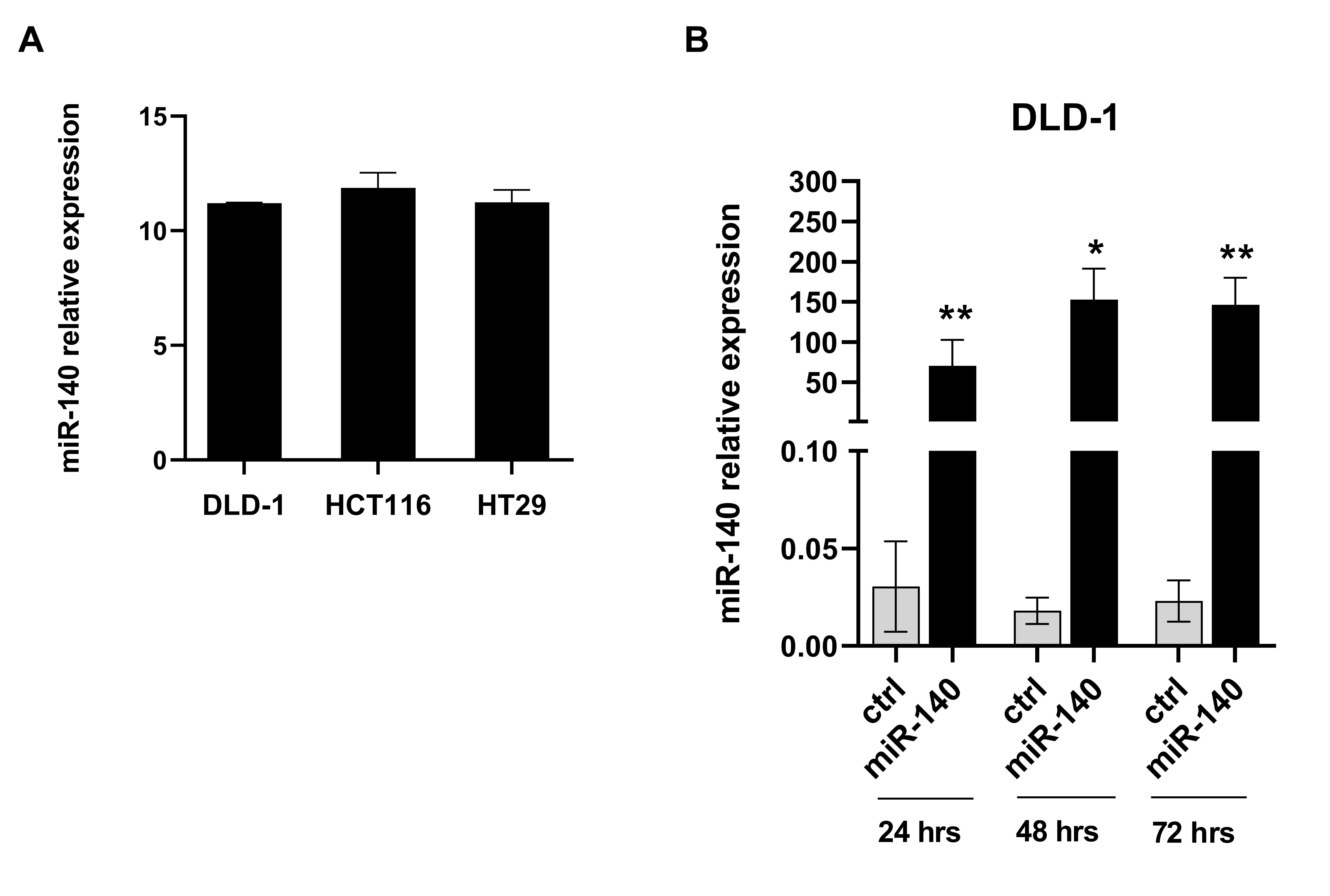

Supplement: Supplementary Figure 1 — (A) relative expression of miR-140 in different colorectal cell lines (B) optimization of the transfection with miR-140 mimics. The results represent the mean value of three independent experiments ± SD *p ≤ 0.05, **p ≤ 0.01, ***p ≤ 0.001 [file Image_1.jpeg]
