# Supplementary material for: MiR-140 leads to MRE11 downregulation and ameliorates oxaliplatin treatment and therapy response in colorectal cancer patients
Source: Front Oncol. 2022 Oct 17;12:959407. doi: 10.3389/fonc.2022.959407 (PMC9618941; doi:10.3389/fonc.2022.959407)
Supplement: Supplementary file 2 [file DataSheet_1.docx]

Supplementary Tab.1 Expanded patients’ characterization

| ID | Age at diagnosis | Sex | Smoking habit | Diagnosis | Location | TNM stage | Metastasis | Adjuvant therapy |
| --- | --- | --- | --- | --- | --- | --- | --- | --- |
| 1 | 65 | Male | Smoker | c18.2 | Colon | Stage III | No | FOLFOX |
| 2 | 75 | Female | Non-smoker | c18.4 | Colon | Stage II | Yes | FUFA Mayo, XELODA+Avastin |
| 3 | 66 | Female | Non-smoker | c18.7 | Colon | Stage IV | Yes | FOLFOX; DeGramond + Avastin; Campto+Erbitux (weekly); DeGramont+Erbitux; XELODA |
| 4 | 69 | Male | Ex-smoker | c19 | Sigmoideum | Stage II | Yes | XELODA; FOLFOX; CAMPTO |
| 5 | 68 | Female | Non-smoker | c18.0 | Colon | Stage II | No | XELODA |
| 6 | 72 | Female | Ex-smoker | c18.0 | Colon | Stage III | Yes | XELODA; deGramont; FOLFOX; irinotecan+vectibix; XELODA+Avastin |
| 7 | 53 | Female | Ex-smoker | c18.1 | Colon | Stage IV | Yes | FOLFOX, FOLFIRI |
| 8 | 71 | Male | Smoker | c18.7 | Colon | Stage III | Yes | FOLFOX; FOLFIRI+Avastin |
| 9 | 74 | Female | Non-smoker | c18.4 | Colon | Stage III | Yes | XELODA |
| 10 | 59 | Male | Smoker | c18.2 | Colon | Stage III | Yes | FOLFOX; FOLFIRI+Avastin; XELODA+Avastin |
| 11 | 56 | Male | Ex-smoker | c19 | Sigmoideum | Stage I | Yes | FOLFOX |
| 12 | 66 | Female | Ex-smoker | c19 | Sigmoideum | Stage III | Yes | FOLFOX; XELODA; deGramont+Avastin; |
| 13 | 74 | Male | Smoker | c18.6 | Colon | Stage IV | Yes | FOLFOX; deGramont; XELODA+Avastin |
| 14 | 62 | Female | Ex-smoker | c18.7 | Colon | Stage III | No | FOLFOX |
| 15 | 54 | Male | Smoker | c18.7 | Colon | Stage II | No | XELODA |
| 16 | 55 | Female | Smoker | c18.7 | Colon | Stage IV | Yes | FOLFOX+Avastin; deGramont-XELODA+Avastin |
| 17 | 62 | Male | Smoker | c18.7 | Colon | Stage III | Yes | FOLFOX |
| 18 | 60 | Female | Non-smoker | c18.7 | Colon | Stage II | No | XELODA |
| 19 | 63 | Male | Ex-smoker | c18.7 | Colon | Stage III | No | FOLFOX |
| 20 | 60 | Male | Non-smoker | c19 | Sigmoideum | Stage IV | Yes | FOLFOX, FOLFIRI, XELODA |
| 21 | 78 | Female | Non-smoker | c18.6 | Colon | Stage IV | Yes | XELODA |
| 22 | 61 | Female | Smoker | c18.0 | Colon | Stage III | No | FOLFOX |
| 23 | 68 | Female | Smoker | c18.0 | Colon | Stage IV | No | FOLFOX |
| 24 | 37 | Male | Smoker | c19 | Sigmoideum | Stage II | No | XELODA |
| 25 | 74 | Female | Ex-smoker | c19 | Sigmoideum | Stage III | Yes | XELODA |
| 26 | 47 | Male | Non-smoker | c18.7 | Colon | Stage II | No | XELODA |
| 27 | 64 | Female | Ex-smoker | c18.2 | Colon | Stage II | No | XELODA |
| 28 | 59 | Female | Smoker | c18.7 | Colon | Stage III | No | FOLFOX |
| 29 | 62 | Male | Ex-smoker | c18.4 | Colon | Stage II | No | XELODA |
| 30 | 82 | Male | Ex-smoker | c18.4 | Colon | Stage III | No | XELODA |
| 31 | 68 | Female | Non-smoker | c18.7 | Colon | Stage III | Yes | FOLFOX, deGramont |
| 32 | 52 | Female | Non-smoker | c18.0 | Colon | Stage III | No | FOLFOX |
| 33 | 67 | Male | Non-smoker | c18.0 | Colon | Stage III | No | FOLFOX |
| 34 | 72 | Female | Smoker | c18.2 | Colon | Stage II | No | XELODA |
| 35 | 65 | Female | Non-smoker | c19 | Sigmoideum | Stage II | No | FOLFOX |
| 36 | 73 | Male | Ex-smoker | c18.3 | Colon | Stage IV | Yes | FOLFOX |
| 37 | 60 | Male | Smoker | c18.4 | Colon | Stage III | No | FOLFOX |
| 38 | 65 | Male | Ex-smoker | c18.7 | Colon | Stage III | No | FOLFOX |
| 39 | 66 | Male | Non-smoker | c18.2 | Colon | Stage III | Yes | FOLFOX; FOLFOX+Avastin; Capecitabin/degramont; TAS 3x |
| 40 | 59 | Male | Non-smoker | c18.0 | Colon | Stage I | No | XELODA |
| 41 | 49 | Female | Smoker | c18.2 | Colon | Stage III | No | FOLFOX |
| 42 | 82 | Male | Non-smoker | c18.3 | Colon | Stage IV | Yes | XELODA |
| 43 | 67 | Male | Ex-smoker | c18.0 | Colon | Stage III | Yes | FOLFOX, deGramont 8x; FOLFIRI+avastin; XELODA+Avastin |
| 44 | 72 | Male | Non-smoker | c18.0 | Colon | Stage III | Yes | XELODA, oxaliplatin+tomudex, CAMPTO |
| 45 | 49 | Female | Smoker | c18.7 | Colon | Stage III | No | FOLFOX |
| 46 | 64 | Male | Ex-smoker | c18.4 | Colon | Stage IV | Yes | FOLFOX+Vectibix, FUFA+Vectibix, Vectibix mono, FOLFIRI+Avastin, Avastin+Xeloda, Lonsurf, Stivarga |
| 47 | 54 | Male | Ex-smoker | c18.5 | Colon | Stage III | No | deGramont, FOLFOX |
| 48 | 66 | Female | Smoker | c18.2 | Colon | Stage II | No | XELODA |
| 49 | 72 | Female | Ex-smoker | c18.7 | Colon | Stage II | No | XELODA |
| 50 | 69 | Male | Ex-smoker | c18.0 | Colon | Stage III | Yes | FOLFOX |

Supplementary Tab.2 MiRNAs with MRE11 as a predicted target from Targetscan database

| **database** | **mature_mirna_acc** | **mature_mirna_id** | **target_symbol** | **target_ensembl** | **score** | **type** |
| --- | --- | --- | --- | --- | --- | --- |
| targetscan | MIMAT0019796 | hsa-mir-4700 | MRE11 | ENSG00000020922 | -0.156 | predicted |
| targetscan | MIMAT0004558 | hsa-mir-181a | MRE11 | ENSG00000020922 | -0.156 | predicted |
| targetscan | MIMAT0004763 | hsa-mir-488 | MRE11 | ENSG00000020922 | -0.156 | predicted |
| targetscan | MIMAT0003278 | hsa-mir-610 | MRE11 | ENSG00000020922 | -0.156 | predicted |
| targetscan | MIMAT0019947 | hsa-mir-4783 | MRE11 | ENSG00000020922 | -0.157 | predicted |
| targetscan | MIMAT0009198 | hsa-mir-224 | MRE11 | ENSG00000020922 | -0.157 | predicted |
| targetscan | MIMAT0019841 | hsa-mir-4724 | MRE11 | ENSG00000020922 | -0.157 | predicted |
| targetscan | MIMAT0030986 | hsa-mir-8059 | MRE11 | ENSG00000020922 | -0.157 | predicted |
| targetscan | MIMAT0005930 | hsa-mir-1276 | MRE11 | ENSG00000020922 | -0.157 | predicted |
| targetscan | MIMAT0025475 | hsa-mir-6509 | MRE11 | ENSG00000020922 | -0.158 | predicted |
| targetscan | MIMAT0022258 | hsa-mir-5571 | MRE11 | ENSG00000020922 | -0.158 | predicted |
| targetscan | MIMAT0027455 | hsa-mir-6777 | MRE11 | ENSG00000020922 | -0.158 | predicted |
| targetscan | MIMAT0004586 | hsa-mir-15b | MRE11 | ENSG00000020922 | -0.159 | predicted |
| targetscan | MIMAT0025458 | hsa-mir-6501 | MRE11 | ENSG00000020922 | -0.159 | predicted |
| targetscan | MIMAT0031012 | hsa-mir-8085 | MRE11 | ENSG00000020922 | -0.16 | predicted |
| targetscan | MIMAT0003235 | hsa-mir-570 | MRE11 | ENSG00000020922 | -0.16 | predicted |
| targetscan | MIMAT0026618 | hsa-mir-585 | MRE11 | ENSG00000020922 | -0.161 | predicted |
| targetscan | MIMAT0027391 | hsa-mir-6745 | MRE11 | ENSG00000020922 | -0.161 | predicted |
| targetscan | MIMAT0015088 | hsa-mir-514b | MRE11 | ENSG00000020922 | -0.162 | predicted |
| targetscan | MIMAT0002883 | hsa-mir-514a | MRE11 | ENSG00000020922 | -0.162 | predicted |
| targetscan | MIMAT0018936 | hsa-mir-4423 | MRE11 | ENSG00000020922 | -0.162 | predicted |
| targetscan | MIMAT0022268 | hsa-mir-548as | MRE11 | ENSG00000020922 | -0.163 | predicted |
| targetscan | MIMAT0005885 | hsa-mir-1295a | MRE11 | ENSG00000020922 | -0.163 | predicted |
| targetscan | MIMAT0003250 | hsa-mir-585 | MRE11 | ENSG00000020922 | -0.163 | predicted |
| targetscan | MIMAT0000720 | hsa-mir-376c | MRE11 | ENSG00000020922 | -0.163 | predicted |
| targetscan | MIMAT0000764 | hsa-mir-339 | MRE11 | ENSG00000020922 | -0.164 | predicted |
| targetscan | MIMAT0025854 | hsa-mir-6722 | MRE11 | ENSG00000020922 | -0.164 | predicted |
| targetscan | MIMAT0022923 | hsa-mir-376b | MRE11 | ENSG00000020922 | -0.165 | predicted |
| targetscan | MIMAT0000441 | hsa-mir-9 | MRE11 | ENSG00000020922 | -0.165 | predicted |
| targetscan | MIMAT0003259 | hsa-mir-591 | MRE11 | ENSG00000020922 | -0.165 | predicted |
| targetscan | MIMAT0004658 | hsa-mir-155 | MRE11 | ENSG00000020922 | -0.166 | predicted |
| targetscan | MIMAT0022294 | hsa-mir-1295b | MRE11 | ENSG00000020922 | -0.166 | predicted |
| targetscan | MIMAT0002807 | hsa-mir-491 | MRE11 | ENSG00000020922 | -0.166 | predicted |
| targetscan | MIMAT0019977 | hsa-mir-4799 | MRE11 | ENSG00000020922 | -0.167 | predicted |
| targetscan | MIMAT0025472 | hsa-mir-6508 | MRE11 | ENSG00000020922 | -0.167 | predicted |
| targetscan | MIMAT0016858 | hsa-mir-4306 | MRE11 | ENSG00000020922 | -0.168 | predicted |
| targetscan | MIMAT0019761 | hsa-mir-4677 | MRE11 | ENSG00000020922 | -0.168 | predicted |
| targetscan | MIMAT0026480 | hsa-mir-153 | MRE11 | ENSG00000020922 | -0.168 | predicted |
| targetscan | MIMAT0000455 | hsa-mir-185 | MRE11 | ENSG00000020922 | -0.168 | predicted |
| targetscan | MIMAT0019763 | hsa-mir-4679 | MRE11 | ENSG00000020922 | -0.168 | predicted |
| targetscan | MIMAT0026641 | hsa-mir-1298 | MRE11 | ENSG00000020922 | -0.169 | predicted |
| targetscan | MIMAT0016890 | hsa-mir-4261 | MRE11 | ENSG00000020922 | -0.17 | predicted |
| targetscan | MIMAT0019901 | hsa-mir-4757 | MRE11 | ENSG00000020922 | -0.171 | predicted |
| targetscan | MIMAT0027626 | hsa-mir-6862 | MRE11 | ENSG00000020922 | -0.173 | predicted |
| targetscan |  | hsa-mir-140 | MRE11 | ENSG00000020922 | -0.174 | predicted |
| targetscan | MIMAT0022984 | hsa-mir-5089 | MRE11 | ENSG00000020922 | -0.174 | predicted |
| targetscan | MIMAT0018940 | hsa-mir-4425 | MRE11 | ENSG00000020922 | -0.175 | predicted |
| targetscan | MIMAT0004927 | hsa-mir-708 | MRE11 | ENSG00000020922 | -0.175 | predicted |
| targetscan | MIMAT0016866 | hsa-mir-4315 | MRE11 | ENSG00000020922 | -0.175 | predicted |
| targetscan | MIMAT0022491 | hsa-mir-5698 | MRE11 | ENSG00000020922 | -0.176 | predicted |
| targetscan | MIMAT0019055 | hsa-mir-4518 | MRE11 | ENSG00000020922 | -0.176 | predicted |
| targetscan | MIMAT0016880 | hsa-mir-4259 | MRE11 | ENSG00000020922 | -0.177 | predicted |
| targetscan | MIMAT0005904 | hsa-mir-1253 | MRE11 | ENSG00000020922 | -0.178 | predicted |
| targetscan | MIMAT0019873 | hsa-mir-4742 | MRE11 | ENSG00000020922 | -0.178 | predicted |
| targetscan | MIMAT0000439 | hsa-mir-153 | MRE11 | ENSG00000020922 | -0.178 | predicted |
| targetscan | MIMAT0019896 | hsa-mir-4755 | MRE11 | ENSG00000020922 | -0.179 | predicted |
| targetscan | MIMAT0002177 | hsa-mir-486 | MRE11 | ENSG00000020922 | -0.179 | predicted |
| targetscan | MIMAT0018114 | hsa-mir-3686 | MRE11 | ENSG00000020922 | -0.179 | predicted |
| targetscan | MIMAT0027515 | hsa-mir-6807 | MRE11 | ENSG00000020922 | -0.18 | predicted |
| targetscan | MIMAT0004500 | hsa-mir-26b | MRE11 | ENSG00000020922 | -0.181 | predicted |
| targetscan | MIMAT0001536 | hsa-mir-429 | MRE11 | ENSG00000020922 | -0.181 | predicted |
| targetscan | MIMAT0000617 | hsa-mir-200c | MRE11 | ENSG00000020922 | -0.181 | predicted |
| targetscan | MIMAT0000318 | hsa-mir-200b | MRE11 | ENSG00000020922 | -0.181 | predicted |
| targetscan | MIMAT0019743 | hsa-mir-4667 | MRE11 | ENSG00000020922 | -0.181 | predicted |
| targetscan | MIMAT0004676 | hsa-mir-34b | MRE11 | ENSG00000020922 | -0.183 | predicted |
| targetscan | MIMAT0007883 | hsa-mir-1909 | MRE11 | ENSG00000020922 | -0.185 | predicted |
| targetscan | MIMAT0030994 | hsa-mir-8067 | MRE11 | ENSG00000020922 | -0.186 | predicted |
| targetscan | MIMAT0027640 | hsa-mir-6870 | MRE11 | ENSG00000020922 | -0.187 | predicted |
| targetscan | MIMAT0028119 | hsa-mir-7111 | MRE11 | ENSG00000020922 | -0.187 | predicted |
| targetscan | MIMAT0019695 | hsa-mir-4638 | MRE11 | ENSG00000020922 | -0.191 | predicted |
| targetscan |  | hsa-mir-124 | MRE11 | ENSG00000020922 | -0.192 | predicted |
| targetscan | MIMAT0021122 | hsa-mir-5191 | MRE11 | ENSG00000020922 | -0.192 | predicted |
| targetscan | MIMAT0019856 | hsa-mir-4732 | MRE11 | ENSG00000020922 | -0.194 | predicted |
| targetscan | MIMAT0027381 | hsa-mir-6740 | MRE11 | ENSG00000020922 | -0.194 | predicted |
| targetscan | MIMAT0005588 | hsa-mir-1233 | MRE11 | ENSG00000020922 | -0.195 | predicted |
| targetscan | MIMAT0022293 | hsa-mir-1295b | MRE11 | ENSG00000020922 | -0.196 | predicted |
| targetscan | MIMAT0004807 | hsa-mir-624 | MRE11 | ENSG00000020922 | -0.196 | predicted |
| targetscan | MIMAT0000764 | hsa-mir-339 | MRE11 | ENSG00000020922 | -0.2 | predicted |
| targetscan | MIMAT0026483 | hsa-mir-370 | MRE11 | ENSG00000020922 | -0.2 | predicted |
| targetscan | MIMAT0026740 | hsa-mir-1250 | MRE11 | ENSG00000020922 | -0.202 | predicted |
| targetscan | MIMAT0003234 | hsa-mir-569 | MRE11 | ENSG00000020922 | -0.202 | predicted |
| targetscan | MIMAT0025454 | hsa-mir-6500 | MRE11 | ENSG00000020922 | -0.203 | predicted |
| targetscan | MIMAT0015049 | hsa-mir-1193 | MRE11 | ENSG00000020922 | -0.203 | predicted |
| targetscan | MIMAT0019690 | hsa-mir-4633 | MRE11 | ENSG00000020922 | -0.203 | predicted |
| targetscan | MIMAT0022288 | hsa-mir-5586 | MRE11 | ENSG00000020922 | -0.203 | predicted |
| targetscan | MIMAT0019766 | hsa-mir-4681 | MRE11 | ENSG00000020922 | -0.204 | predicted |
| targetscan | MIMAT0019872 | hsa-mir-4742 | MRE11 | ENSG00000020922 | -0.208 | predicted |
| targetscan | MIMAT0005881 | hsa-mir-1291 | MRE11 | ENSG00000020922 | -0.208 | predicted |
| targetscan | MIMAT0015000 | hsa-mir-3134 | MRE11 | ENSG00000020922 | -0.209 | predicted |
| targetscan | MIMAT0016846 | hsa-mir-4297 | MRE11 | ENSG00000020922 | -0.209 | predicted |
| targetscan |  | hsa-mir-1912 | MRE11 | ENSG00000020922 | -0.21 | predicted |
| targetscan | MIMAT0002868 | hsa-mir-522 | MRE11 | ENSG00000020922 | -0.212 | predicted |
| targetscan | MIMAT0004678 | hsa-mir-99b | MRE11 | ENSG00000020922 | -0.212 | predicted |
| targetscan | MIMAT0004511 | hsa-mir-99a | MRE11 | ENSG00000020922 | -0.212 | predicted |
| targetscan | MIMAT0024611 | hsa-mir-6128 | MRE11 | ENSG00000020922 | -0.213 | predicted |
| targetscan | MIMAT0005929 | hsa-mir-1275 | MRE11 | ENSG00000020922 | -0.214 | predicted |
| targetscan | MIMAT0018188 | hsa-mir-3914 | MRE11 | ENSG00000020922 | -0.214 | predicted |
| targetscan | MIMAT0019220 | hsa-mir-3664 | MRE11 | ENSG00000020922 | -0.214 | predicted |
| targetscan | MIMAT0000683 | hsa-mir-302a | MRE11 | ENSG00000020922 | -0.215 | predicted |
| targetscan | MIMAT0023711 | hsa-mir-6086 | MRE11 | ENSG00000020922 | -0.216 | predicted |
| targetscan | MIMAT0019838 | hsa-mir-4723 | MRE11 | ENSG00000020922 | -0.22 | predicted |
| targetscan | MIMAT0016873 | hsa-mir-4322 | MRE11 | ENSG00000020922 | -0.221 | predicted |
| targetscan | MIMAT0027398 | hsa-mir-6749 | MRE11 | ENSG00000020922 | -0.222 | predicted |
| targetscan | MIMAT0015078 | hsa-mir-3194 | MRE11 | ENSG00000020922 | -0.223 | predicted |
| targetscan | MIMAT0019751 | hsa-mir-4670 | MRE11 | ENSG00000020922 | -0.223 | predicted |
| targetscan | MIMAT0004557 | hsa-mir-34a | MRE11 | ENSG00000020922 | -0.224 | predicted |
| targetscan | MIMAT0016845 | hsa-mir-4296 | MRE11 | ENSG00000020922 | -0.225 | predicted |
| targetscan | MIMAT0030996 | hsa-mir-8069 | MRE11 | ENSG00000020922 | -0.225 | predicted |
| targetscan | MIMAT0002874 | hsa-mir-503 | MRE11 | ENSG00000020922 | -0.228 | predicted |
| targetscan | MIMAT0019023 | hsa-mir-4489 | MRE11 | ENSG00000020922 | -0.228 | predicted |
| targetscan | MIMAT0019878 | hsa-mir-4745 | MRE11 | ENSG00000020922 | -0.228 | predicted |
| targetscan | MIMAT0019916 | hsa-mir-4765 | MRE11 | ENSG00000020922 | -0.232 | predicted |
| targetscan | MIMAT0015022 | hsa-mir-3149 | MRE11 | ENSG00000020922 | -0.232 | predicted |
| targetscan | MIMAT0027490 | hsa-mir-6795 | MRE11 | ENSG00000020922 | -0.233 | predicted |
| targetscan | MIMAT0003261 | hsa-mir-593 | MRE11 | ENSG00000020922 | -0.233 | predicted |
| targetscan |  | hsa-mir-1912 | MRE11 | ENSG00000020922 | -0.233 | predicted |
| targetscan | MIMAT0025850 | hsa-mir-6719 | MRE11 | ENSG00000020922 | -0.233 | predicted |
| targetscan | MIMAT0027451 | hsa-mir-6775 | MRE11 | ENSG00000020922 | -0.234 | predicted |
| targetscan | MIMAT0022275 | hsa-mir-5581 | MRE11 | ENSG00000020922 | -0.235 | predicted |
| targetscan | MIMAT0024611 | hsa-mir-6128 | MRE11 | ENSG00000020922 | -0.235 | predicted |
| targetscan | MIMAT0004689 | hsa-mir-377 | MRE11 | ENSG00000020922 | -0.235 | predicted |
| targetscan | MIMAT0027631 | hsa-mir-6865 | MRE11 | ENSG00000020922 | -0.236 | predicted |
| targetscan | MIMAT0031016 | hsa-mir-8089 | MRE11 | ENSG00000020922 | -0.237 | predicted |
| targetscan | MIMAT0004605 | hsa-mir-129 | MRE11 | ENSG00000020922 | -0.238 | predicted |
| targetscan | MIMAT0004548 | hsa-mir-129 | MRE11 | ENSG00000020922 | -0.238 | predicted |
| targetscan | MIMAT0018091 | hsa-mir-3668 | MRE11 | ENSG00000020922 | -0.239 | predicted |
| targetscan | MIMAT0027479 | hsa-mir-6789 | MRE11 | ENSG00000020922 | -0.239 | predicted |
| targetscan |  | hsa-mir-203a | MRE11 | ENSG00000020922 | -0.241 | predicted |
| targetscan | MIMAT0019869 | hsa-mir-4740 | MRE11 | ENSG00000020922 | -0.243 | predicted |
| targetscan | MIMAT0019059 | hsa-mir-1269b | MRE11 | ENSG00000020922 | -0.244 | predicted |
| targetscan | MIMAT0003294 | hsa-mir-625 | MRE11 | ENSG00000020922 | -0.244 | predicted |
| targetscan | MIMAT0005923 | hsa-mir-1269a | MRE11 | ENSG00000020922 | -0.244 | predicted |
| targetscan | MIMAT0000750 | hsa-mir-340 | MRE11 | ENSG00000020922 | -0.244 | predicted |
| targetscan | MIMAT0027461 | hsa-mir-6780a | MRE11 | ENSG00000020922 | -0.245 | predicted |
| targetscan | MIMAT0003328 | hsa-mir-653 | MRE11 | ENSG00000020922 | -0.245 | predicted |
| targetscan | MIMAT0022293 | hsa-mir-1295b | MRE11 | ENSG00000020922 | -0.246 | predicted |
| targetscan | MIMAT0002872 | hsa-mir-501 | MRE11 | ENSG00000020922 | -0.246 | predicted |
| targetscan | MIMAT0027663 | hsa-mir-6881 | MRE11 | ENSG00000020922 | -0.247 | predicted |
| targetscan | MIMAT0028220 | hsa-mir-7155 | MRE11 | ENSG00000020922 | -0.248 | predicted |
| targetscan | MIMAT0022271 | hsa-mir-664b | MRE11 | ENSG00000020922 | -0.248 | predicted |
| targetscan | MIMAT0001618 | hsa-mir-191 | MRE11 | ENSG00000020922 | -0.249 | predicted |
| targetscan | MIMAT0015007 | hsa-mir-3139 | MRE11 | ENSG00000020922 | -0.25 | predicted |
| targetscan | MIMAT0026734 | hsa-mir-942 | MRE11 | ENSG00000020922 | -0.251 | predicted |
| targetscan | MIMAT0022725 | hsa-mir-1255b | MRE11 | ENSG00000020922 | -0.253 | predicted |
| targetscan | MIMAT0016891 | hsa-mir-4265 | MRE11 | ENSG00000020922 | -0.254 | predicted |
| targetscan | MIMAT0022271 | hsa-mir-664b | MRE11 | ENSG00000020922 | -0.255 | predicted |
| targetscan | MIMAT0027674 | hsa-mir-6887 | MRE11 | ENSG00000020922 | -0.255 | predicted |
| targetscan | MIMAT0019961 | hsa-mir-4790 | MRE11 | ENSG00000020922 | -0.257 | predicted |
| targetscan | MIMAT0000085 | hsa-mir-28 | MRE11 | ENSG00000020922 | -0.26 | predicted |
| targetscan | MIMAT0005907 | hsa-mir-1256 | MRE11 | ENSG00000020922 | -0.262 | predicted |
| targetscan | MIMAT0005920 | hsa-mir-1266 | MRE11 | ENSG00000020922 | -0.265 | predicted |
| targetscan | MIMAT0014990 | hsa-mir-3127 | MRE11 | ENSG00000020922 | -0.268 | predicted |
| targetscan | MIMAT0015072 | hsa-mir-320e | MRE11 | ENSG00000020922 | -0.272 | predicted |
| targetscan | MIMAT0004926 | hsa-mir-708 | MRE11 | ENSG00000020922 | -0.276 | predicted |
| targetscan | MIMAT0032116 | hsa-mir-4485 | MRE11 | ENSG00000020922 | -0.277 | predicted |
| targetscan | MIMAT0027555 | hsa-mir-6827 | MRE11 | ENSG00000020922 | -0.278 | predicted |
| targetscan | MIMAT0019739 | hsa-mir-4665 | MRE11 | ENSG00000020922 | -0.279 | predicted |
| targetscan | MIMAT0019080 | hsa-mir-4537 | MRE11 | ENSG00000020922 | -0.281 | predicted |
| targetscan | MIMAT0004450 | hsa-mir-297 | MRE11 | ENSG00000020922 | -0.287 | predicted |
| targetscan | MIMAT0031000 | hsa-mir-8073 | MRE11 | ENSG00000020922 | -0.29 | predicted |
| targetscan | MIMAT0019908 | hsa-mir-4761 | MRE11 | ENSG00000020922 | -0.294 | predicted |
| targetscan | MIMAT0015031 | hsa-mir-3157 | MRE11 | ENSG00000020922 | -0.295 | predicted |
| targetscan | MIMAT0004568 | hsa-mir-221 | MRE11 | ENSG00000020922 | -0.298 | predicted |
| targetscan | MIMAT0015065 | hsa-mir-3185 | MRE11 | ENSG00000020922 | -0.301 | predicted |
| targetscan | MIMAT0005912 | hsa-mir-548g | MRE11 | ENSG00000020922 | -0.301 | predicted |
| targetscan | MIMAT0000227 | hsa-mir-197 | MRE11 | ENSG00000020922 | -0.304 | predicted |
| targetscan | MIMAT0027596 | hsa-mir-6848 | MRE11 | ENSG00000020922 | -0.306 | predicted |
| targetscan | MIMAT0027592 | hsa-mir-6846 | MRE11 | ENSG00000020922 | -0.306 | predicted |
| targetscan | MIMAT0022725 | hsa-mir-1255b | MRE11 | ENSG00000020922 | -0.316 | predicted |
| targetscan | MIMAT0027493 | hsa-mir-6796 | MRE11 | ENSG00000020922 | -0.316 | predicted |
| targetscan | MIMAT0019949 | hsa-mir-4785 | MRE11 | ENSG00000020922 | -0.325 | predicted |
| targetscan | MIMAT0016877 | hsa-mir-4256 | MRE11 | ENSG00000020922 | -0.329 | predicted |
| targetscan | MIMAT0027361 | hsa-mir-6730 | MRE11 | ENSG00000020922 | -0.335 | predicted |
| targetscan | MIMAT0004917 | hsa-mir-888 | MRE11 | ENSG00000020922 | -0.356 | predicted |
| targetscan | MIMAT0004814 | hsa-mir-654 | MRE11 | ENSG00000020922 | -0.356 | predicted |
| targetscan | MIMAT0015061 | hsa-mir-3181 | MRE11 | ENSG00000020922 | -0.361 | predicted |
| targetscan | MIMAT0027510 | hsa-mir-6805 | MRE11 | ENSG00000020922 | -0.363 | predicted |
| targetscan | MIMAT0018003 | hsa-mir-3622a | MRE11 | ENSG00000020922 | -0.367 | predicted |
| targetscan | MIMAT0019719 | hsa-mir-4653 | MRE11 | ENSG00000020922 | -0.372 | predicted |
| targetscan | MIMAT0018925 | hsa-mir-1268b | MRE11 | ENSG00000020922 | -0.378 | predicted |
| targetscan | MIMAT0005922 | hsa-mir-1268a | MRE11 | ENSG00000020922 | -0.378 | predicted |
| targetscan | MIMAT0027472 | hsa-mir-6786 | MRE11 | ENSG00000020922 | -0.389 | predicted |
| targetscan | MIMAT0028214 | hsa-mir-7152 | MRE11 | ENSG00000020922 | -0.406 | predicted |
| targetscan | MIMAT0016874 | hsa-mir-4321 | MRE11 | ENSG00000020922 | -0.414 | predicted |
| targetscan | MIMAT0022485 | hsa-mir-4666b | MRE11 | ENSG00000020922 | -0.431 | predicted |
| targetscan | MIMAT0025484 | hsa-mir-6514 | MRE11 | ENSG00000020922 | -0.439 | predicted |
| targetscan | MIMAT0022975 | hsa-mir-3934 | MRE11 | ENSG00000020922 | -0.494 | predicted |

Supplementary Tab.3 Progression free survival analysis of predicted miRNAs on TCGA samples

| **MiRNA_ID** | **Progression_free_survival_logrank_p** | **Number_of_patients** |
| --- | --- | --- |
| hsa-mir-7155 | 0,00398829 | 275 |
| hsa-mir-140 | 0,006077677 | 570 |
| hsa-mir-6514 | 0,00714145 | 301 |
| hsa-mir-200c | 0,016336796 | 570 |
| hsa-mir-4306 | 0,025699082 | 40 |
| hsa-mir-221 | 0,034546413 | 570 |
| hsa-mir-224 | 0,039636068 | 570 |
| hsa-mir-3127 | 0,045066369 | 561 |
| hsa-mir-942 | 0,055731924 | 569 |
| hsa-mir-5581 | 0,057226548 | 276 |
| hsa-mir-200b | 0,062337996 | 570 |
| hsa-mir-4724 | 0,069961248 | 458 |
| hsa-mir-501 | 0,098223307 | 570 |
| hsa-mir-34b | 0,09921315 | 417 |
| hsa-mir-6749 | 0,119865266 | 68 |
| hsa-mir-664b | 0,129438748 | 548 |
| hsa-mir-664b | 0,129438748 | 548 |
| hsa-mir-5698 | 0,132430706 | 358 |
| hsa-mir-185 | 0,143193758 | 570 |
| hsa-mir-4742 | 0,163728433 | 380 |
| hsa-mir-4742 | 0,163728433 | 380 |
| hsa-mir-488 | 0,166650554 | 38 |
| hsa-mir-3934 | 0,17118315 | 548 |
| hsa-mir-4740 | 0,176016898 | 71 |
| hsa-mir-99a | 0,197317565 | 570 |
| hsa-mir-7111 | 0,19892365 | 56 |
| hsa-mir-6501 | 0,20069312 | 235 |
| hsa-mir-1909 | 0,218821575 | 112 |
| hsa-mir-4425 | 0,221645633 | 45 |
| hsa-mir-514b | 0,230527259 | 116 |
| hsa-mir-1291 | 0,233276437 | 486 |
| hsa-mir-34a | 0,240119494 | 570 |
| hsa-mir-191 | 0,240408238 | 570 |
| hsa-mir-4785 | 0,247982911 | 113 |
| hsa-mir-1295a | 0,257521828 | 263 |
| hsa-mir-570 | 0,3001149 | 453 |
| hsa-mir-1295b | 0,305623203 | 67 |
| hsa-mir-1295b | 0,305623203 | 67 |
| hsa-mir-1295b | 0,305623203 | 67 |
| hsa-mir-3157 | 0,325688272 | 356 |
| hsa-mir-6827 | 0,336592037 | 55 |
| hsa-mir-5571 | 0,339012419 | 206 |
| hsa-mir-522 | 0,339816613 | 93 |
| hsa-mir-6786 | 0,349828677 | 52 |
| hsa-mir-6789 | 0,358612051 | 52 |
| hsa-mir-1266 | 0,391916474 | 567 |
| hsa-mir-3139 | 0,403230951 | 44 |
| hsa-mir-4638 | 0,405838134 | 453 |
| hsa-mir-4665 | 0,414198367 | 239 |
| hsa-mir-320e | 0,418639902 | 105 |
| hsa-mir-3194 | 0,431653877 | 343 |
| hsa-mir-585 | 0,435025326 | 262 |
| hsa-mir-585 | 0,435025326 | 262 |
| hsa-mir-1275 | 0,4458372 | 356 |
| hsa-mir-4653 | 0,451297773 | 66 |
| hsa-mir-625 | 0,454756259 | 570 |
| hsa-mir-15b | 0,483553421 | 570 |
| hsa-mir-1269b | 0,501355514 | 214 |
| hsa-mir-26b | 0,501567685 | 570 |
| hsa-mir-8059 | 0,503494396 | 65 |
| hsa-mir-4755 | 0,528823503 | 220 |
| hsa-mir-99b | 0,536755193 | 570 |
| hsa-mir-4723 | 0,538995222 | 59 |
| hsa-mir-624 | 0,56156972 | 506 |
| hsa-mir-1256 | 0,573570598 | 140 |
| hsa-mir-3622a | 0,623772073 | 88 |
| hsa-mir-6740 | 0,642050207 | 73 |
| hsa-mir-3664 | 0,655179444 | 426 |
| hsa-mir-203a | 0,665874985 | 570 |
| hsa-mir-4757 | 0,695703325 | 61 |
| hsa-mir-376b | 0,71196985 | 523 |
| hsa-mir-503 | 0,717412201 | 570 |
| hsa-mir-1298 | 0,730976001 | 31 |
| hsa-mir-6777 | 0,771533734 | 191 |
| hsa-mir-6887 | 0,774971458 | 135 |
| hsa-mir-339 | 0,775366495 | 570 |
| hsa-mir-339 | 0,775366495 | 570 |
| hsa-mir-6775 | 0,787321506 | 51 |
| hsa-mir-6805 | 0,788849549 | 130 |
| hsa-mir-6509 | 0,794938148 | 378 |
| hsa-mir-6870 | 0,798923398 | 91 |
| hsa-mir-1269a | 0,801772529 | 504 |
| hsa-mir-4423 | 0,804601228 | 153 |
| hsa-mir-1276 | 0,81886676 | 245 |
| hsa-mir-6730 | 0,838480417 | 169 |
| hsa-mir-6508 | 0,843030427 | 89 |
| hsa-mir-6846 | 0,844514748 | 36 |
| hsa-mir-376c | 0,846410605 | 560 |
| hsa-mir-1250 | 0,849815959 | 83 |
| hsa-mir-4700 | 0,852758307 | 97 |
| hsa-mir-155 | 0,873236813 | 570 |
| hsa-mir-708 | 0,87749198 | 570 |
| hsa-mir-708 | 0,87749198 | 570 |
| hsa-mir-4667 | 0,888232397 | 79 |
| hsa-mir-4732 | 0,895406318 | 204 |
| hsa-mir-370 | 0,897145069 | 569 |
| hsa-mir-28 | 0,901785459 | 570 |
| hsa-mir-340 | 0,914707481 | 570 |
| hsa-mir-654 | 0,920218606 | 570 |
| hsa-mir-429 | 0,927688972 | 570 |
| hsa-mir-377 | 0,933002231 | 559 |
| hsa-mir-3149 | 0,935098624 | 59 |
| hsa-mir-197 | 0,937457491 | 570 |
| hsa-mir-653 | 0,940606788 | 558 |
| hsa-mir-4745 | 0,966481202 | 184 |
| hsa-mir-4677 | 0,974596226 | 568 |
| hsa-mir-491 | 0,97977349 | 556 |
| hsa-mir-6807 | 0,982927835 | 131 |
| hsa-mir-4799 | 0,989964188 | 31 |
| hsa-mir-6796 | 0,998808455 | 39 |
| hsa-mir-5586 | 0,998948833 | 511 |
